# Development of a cell-based high throughput assay system and IN-house image analysis software for screening of active compounds against dengue virus

**DOI:** 10.1186/1753-6561-5-S1-P46

**Published:** 2011-01-10

**Authors:** Deu John M Cruz, Andrea C Koishi, Ana Luiza M Pamplona, Auguste Genovesio, Claudia N Duarte dos Santos, Lucio Freitas-Junior

**Affiliations:** 1 Center for Neglected Diseases Drug Discovery (CND3) – Dengue Drug Discovery, Institut Pasteur Korea, Seongnam-si, 463-400, Korea; 2 Carlos-Chagas Institute- FIOCRUZ, Curitiba, 81350-010 Brazil; 3 Post-Graduate Programm in Molecular and Cell Biology, Universidade Federal do Parana, Curitiba 81530-130, Brazil; 4 Center for Core Technologies-Image Mining, Institut Pasteur Korea, Seongnam-si, 463-400, Korea

## 

Dengue virus (DV) is a mosquito-borne pathogen capable of infecting multiple target organs in the human host. Infection with any one of the four dengue serotypes can cause an acute febrile illness known as Dengue Fever (DF), while subsequent infection with a heterologous serotype or a highly virulent strain can result in the more severe and sometimes lethal Dengue Hemorrhagic Fever (DHF) and Dengue Shock Syndrome (DSS). According to the World Health Organization, approximately 2.5 billion people live in dengue endemic countries, with an estimated 50 million cases occurring annually. The lack of an effective dengue vaccine has prompted the need to discover compounds that can inhibit DV infection. In this study, a cell-based, high-throughput assay system that utilizes an image-based analysis algorithm for evaluating active compounds that target DV infection is described. Using representative strains of DV1, DV2, DV3 serotypes, a human-derived hepatoma cell line (Huh7.5) and *Aedes albopictus* mosquito-derived cell line (clone C6/36) were inoculated with a high titer of each virus in a 384-well plate culture system in the presence of varying concentration of known active compounds. After 1-2 rounds of viral replication (48~72 h), infection was arrested by para-formaldehyde fixation. DV-infected cells were visualized by probing with D1-4G2-4-15 mAb, a flavivirus group-specific monoclonal antibody that targets the E protein, and a mouse IgG-specific AlexaFluor488™ secondary antibody. Images of the DV-infected cell culture are captured with an automated confocal microscope (Evotec Opera™), and analyzed using IM v3.0, a custom-based image analysis software developed by IP-Korea’s Image Mining Group. A 10-point dose-response curve was generated for each active compound and reproduced in several experiments. This newly developed HTA (High Throughput Assay) system for Dengue can be a useful tool to screen large compound libraries for active drugs that have inhibitory effects to DV infection.

